# Genomics unveils country-to-country transmission between animal hospitals of a multidrug-resistant and sequence type 2 Acinetobacter baumannii clone

**DOI:** 10.1099/mgen.0.001292

**Published:** 2024-10-14

**Authors:** Amédée André, Julie Plantade, Isabelle Durieux, Pauline Durieu, Anne-Sophie Godeux, Maxence Decellieres, Céline Pouzot-Nevoret, Samuel Venner, Xavier Charpentier, Maria-Halima Laaberki

**Affiliations:** 1 CIRI, Centre International de Recherche en Infectiologie, Inserm, U1111, Université Claude Bernard Lyon 1, CNRS, UMR5308, École Normale Supérieure de Lyon, Univ Lyon, 69007 Lyon, France; 2 Université de Lyon, VeAgro Sup, 69280 Marcy l'Etoile, France; 3 Université de Lyon, VeAgro Sup, Intensive Care Unit (SIAMU), APCSe, 69280, Marcy l'Etoile, France; 4 UMR CNRS 5558 – LBBE 'Laboratoire de Biométrie et Biologie Évolutive', Université Claude Bernard Lyon 1, Villeurbanne, France

**Keywords:** *Acinetobacter baumannii*, antibiotic resistance, resistance island, zoonosis

## Abstract

*Acinetobacter baumannii* is a globally distributed opportunistic pathogen in human health settings, including in intensive care units (ICUs). We investigated the contamination of a French small animal ICU with *A. baumannii*. We discovered repeated animal contamination by *A. baumannii*, and phylogenetic analysis traced contamination back to a potential foreign animal origin. Genomic analysis combined with antibiotic susceptibility testing revealed heteroresistance to penicillin and aminoglycoside mediated by insertion sequence dynamics and also suggest a potential cross-resistance to human-restricted piperacillin–tazobactam combination. The *A. baumannii* isolates of the animal ICU belong to the International Clone 2 commonly found in human health settings. Our results suggest a high adaptation of this lineage to healthcare settings and provide questions on the requirements for genetic determinants enabling adaptation to host and abiotic conditions.

## Data Summary

The newly sequenced genomes are listed in Table S1 (available in the online version of this article); these genomes have been submitted to the NCBI and their BioProject number is PRJNA1023193. The publicly available human and animal genomes used in this study are listed in Table S2. All the supplementary tables are available in the Supplementary Material File.

**Impact Statement**

Antibiotic resistance is a global threat affecting both human and animal health. Given the interconnections between these health sectors, the fight against antimicrobial resistance cannot be envisaged in silos, and requires an understanding of the dynamics and transmission mechanisms of multidrug-resistant bacteria. Our study investigates the contamination of an intensive care unit in a veterinary university hospital by the nosocomial and opportunistic agent *Acinetobacter baumannii*. Based on whole genome sequencing of the strains resulting from this investigation, we were able to highlight recurrent contamination by a clone of the International Clone 2 lineage, which dominates epidemiology in human health. By comparing these genomes with genomic databases, we have been able to retrace a potential foreign origin of this clone. These results raise questions about the means by which this clone is transferred and persists between veterinary hospitals, and more broadly about the zoonotic nature of antibiotic resistance.

## Introduction

*Acinetobacter baumannii* is responsible of healthcare-associated infections in both human and animal patients. Rapid acquisition of multidrug resistance, metabolic versatility and survival in hospital settings lead to hospital outbreaks that are difficult to manage. In the human sector, the mortality rate associated with *A. baumannii* infection can greatly vary from 16 to 76% with multidrug-resistant strains infecting patients being associated with poor outcomes [1,3]. Epidemiology in the human sector is dominated by a few lineages shared worldwide with strains of predominantly the International Clones 2 and 5 (IC2 and 5) [4]. In all major globally distributed clones, antibiotic resistance is mainly due to large chromosomal resistance islands such as AbGRI islands found in the IC2 lineage [5,7]. Genomic epidemiology has been used to study the lineages or clones in circulation in different hospitals worldwide. Although repeated local emergences and diffusion of new clones such as ST^Pas^499, ST^Pas^25 or ST^Pas^85 are regularly observed, they fail to replace dominant clones [2,8, 9]. This suggests that IC2, and more particularly the ST2 clade, is well fitted to human populations and/or hospital environments. Consequently, publicly available *A. baumannii* genomes are mostly from this lineage. These genomes are related to human medicine and mainly isolated from infection and to a lesser extent the hospital environment and patient carriage screening. In the animal sector, data are scarce. However, the animal sector involves a great diversity of animals and habitats from wild animals to domestic animals living in close proximity to humans such as pets. For this latter animal category, studies investigating *A. baumannii* lineages in veterinary clinical samples indicate that animal infection mainly involves lineages found in human medicine such as IC1, IC2 and ST25 [10,12]. This underlines the porous nature of human and companion animal health, as well as the similarities between therapeutic approaches. However, antibiotic prescription differs with antibiotics restricted to human medicine such as carbapenems or piperacillin associated with tazobactam [13]. However, pets’ carriage and infection with *A. baumannii* resistant to carbapenems has been described [14]. This underlines the need to perform genomic epidemiology in the companion animal health sector to understand the route of contamination and role of antibiotic treatment in resistance diffusion [15]. In this study, we analyse the contamination with *A. baumannii* of pets during hospitalization, investigate their potential origin and analyze their aresenal of antibiotic resistance over time.

## Methods

### Study design and bacterial strains

This study design was approved by VetAgro Sup ethic committee approval no. 2078. Between April 2021 and April 2022, 94 pets [dogs (*n*=60) and cats (*n*=34)] were sampled in the same intensive care unit (ICU) of the hospital at the veterinary medicine school of Lyon in France (SIAMU, VetAgro Sup, Marcy l'étoile). Informed consent from owners to sample their animal was obtained prior to recruitment. Animals were sampled once during hospitalization, between 1 and 13 days following admission. All animals were subjected to oral swabbing by rubbing a swab on the inner cheek (Amies Agar M40 w/o charcoal, COPAN). The swabs were kept at room temperature in the transport medium overnight before plating on selective medium. The selective medium was CHROMagar Acinetobacter (CHROMagar) that was prepared according to the manufacturer’s instructions without addition of the CHROMagar MDR supplement CR102. Environmental sampling of the ICU was performed using sampling wipes [smooth 30×17 cm sterilized buffered peptone water – Neutralizer (at 10%)] on various fomites. Each wipe was placed in a sterile stomacher bag. Further processing was performed by adding 5 ml of minimal medium with acetate and vortexing; this was left for 5 h at 37 °C with shaking and then 200 µl was spread on the selective medium. Colonies morphologically similar to *A. baumannii* were subjected to PCR identification using primer annealing on the *oxa-51* gene according to previous work [16] with one isolate per animal with the exception of one animal (Dog#7) for which two morphologically distinct colonies were characterized. A list of strains is given in Table S1.

### Genome sequencing and analysis

Four-hour cultures were grown in LB liquid medium and DNA was isolated using a Wizard genomic DNA kit (Promega). For each isolate, both Oxford Nanopore and Illumina DNA sequencing were performed. Nanopore sequencing were performed using MinIon flow cells (R10.4.1). Prior to nanopore barcoding (native barcoding SQK-NBD112.24), DNA quantification was performed using a Qubit dsDNA HS Assay Kit (Thermo Fisher Scientific). Basecalling was performed using ONT Guppy basecalling software v.6.0.6+8a98bbcbd, and reads were filtered out by this software when their quality was less than 10. Illumina sequencing was performed on an Illumina HiSeq 2000 with paired 150-base sequence reads (Novogene). A quality check was performed on Illumina reads using fastqc (0.11.9) [17], and none of the sequences required trimming. Assemblies were performed using Unicycler (v.0.4.9). Quality checking was performed on the 14 hybrid assemblies using CheckM (v.1.2.3) [18], using the flag --reduced_tree to avoid memory issues. All assemblies displayed a completeness of 100%. Twelve assemblies had no trace of contamination, while ABO21-A001 and ABO21-A049 had a percentage of contamination below 5% (0.143 and 0.574% respectively). All genomes are available at NCBI under BioProject PRJNA1023193. Genome accession numbers are listed in Table S1.

For analysis purposes, genomes were annotated using Prokka software (v.1.14.5) on the Galaxy pipeline using as reference AB5075-UW protein sequences (GenBank accession number: CP008706.1). Putative resistance genes were detected using Resfinder [19] and insertion sequences using ISfinder [20]. Strain typing using the Pasteur and Oxford scheme was performed by submitting the genomes to PubMLST [21]. Genomic comparisons were performed and illustrated using GenoFig (https://forgemia.inra.fr/public-pgba/genofig). Plasmid typing was performed using a published typing database [22]. Plasmid presence was verified by performing small-scale plasmid extraction (Omega- Bio-tek), and plasmids were analysed after migration on 1% agarose gel buffered with Tris-acetate followed by ethidium bromide staining. Variant detection analysis was performed using SNIPPY [23].

### Identification of closest related genomes

#### Construction of a 4325 isolate database

In order to build a panallelome, i.e. a local database with protein sequences of *A. baumannii* strains available on NCBI, 4325 GenBank accession numbers were retrieved from NCBI on 17 February 2023. These GenBank accession numbers were identified through the NCBI Genome database, with the following search criteria – key words: *Acinetobacter baumannii*, assembly level: chromosome, complete and contig, exclude partial, exclude anomalous. The protein sequences were then passed to WhatsGNU (v.1.0) [24] to build the panallelome with the script WhatsGNU_GenBank_genomes.py, and the protein identifiers were customized (i.e. the strain name was added to the protein identifiers) using the script WhastGNU_database_customizer.py.

#### Identification of top genomes

Each of the 14 animal ICU isolates was compared to the panallelome using the script WhatsGNU_main.py, with the argument -t (top genome utility) to retrieve the top 10 protein matching strains in the panallelome. The top genome names of all 14 isolates were then concatenated and duplicates were removed using the unique() function in R base. The final top genomes were selected based on three considerations: the number of protein matches (roughly, the top genomes were conserved when the number of matches was above 2500), ST Pasteur and ST Oxford. Overall, seven top genomes were selected for core gene multilocus sequence typing (cgMLST) and phylogeny (MS14413, KAB03, Cl415, ORAB01, AF-673, BAL062 and XH386).

### Core genome multi locus sequence typing

In total, fasta files of 140 RefSeq genomes (134 isolates retrieved from the supplementary materials of Mateo-Estrada *et al*. [25] and six top genomes) were retrieved from the NCBI database, using the NCBI Datasets command line tool (ncbi_datasets, v.14.19.0). The list of strains with their RefSeq accession number, location, host, ST Pasteur and ST Oxford is available in Table S2. cgMLST was performed on the 14 ABO21 strains and the 140 NCBI strains, with chewBBACA (v.3.3.1) [26]. The training file used to build the cgMLST schema is available on github (https://github.com/B-UMMI/chewBBACA, Acinetobacter_baumannii.trn, retrieved 26 July 2022). The schema comprised 2390 loci. The maximum spanning tree was built based on the cgMLST determined by chewBBACA, using MSTreeV2 implemented in grapetree (v.2.2) [27].

### IC2 maximum likelihood phylogeny

A recombination-free alignment of 98 selected isolates was built using SKA2 (0.3.5) implemented in the generate_ska_alignment.py script of gubbins (3.3.1), with default parameters [28]. The alignment was then given to the run_gubbins.py script. To find the best fitting tree, five independent runs of the script were launched, with a different manually fixed seed for each run (argument --seed). The five different seeds were randomly generated using the R command sample (1 : 1 000 000, 5). Each tree was built with RAxML under a GTR model, and 10^3^ bootstraps were generated. The five resulting trees were then compared using the treedist function from the phangorn package (2.11.1). As no difference was identified between the five trees, the first one was selected for visual display (seed=403 202), which was built using the ggtree (3.8.2) and ggnewscale (0.4.10) packages. As we did not conduct molecular clock analysis, and were not able to find a suitable outgroup isolate, the final tree has not been rooted. Besides IC2 study isolates, we included seven isolates identified as the closest genomes via the WhatsGNU top genome utility (MS14413, KAB03, Cl415, ORAB01, AF-673, BAL062 and XH386), four isolates identified as carriers of ABGRI1 (5847, CNRAB1, DETAB-E107 and AB3-VUB), six isolates selected based on their ST Oxford (208 or 350) in the PubMLST database, and three isolates found as collected on dogs and available in the PathogenWatch database. In addition, 68 isolates randomly selected in the PathogenWatch database and displaying an ST Oxford identical to the 30 above-mentioned isolates were included to give a larger perspective of the position of animal ICU isolates in the IC2 phylogeny.

### Gene content analysis

Gene content analysis included the 21 isolates, selected based on their position inside the ST350 (Oxford) clade in the IC2 phylogeny. This clade was defined as all isolates descending from the common ancestor of the nine ABO21 ST350 (Oxford) isolates and NIPH-528 isolate . Their pangenome was built based on assemblies of the 12 non-ABO21 isolates annotated with prokka (via conda) (1.14.6) [29] and using strain 40 288 as reference (GCA_019457715.1). Annotation files (prokka gff) were subjected to Roary (3.13.0) [30]. Roary’s output file ‘gene_presence_absence.Rtab’ was used to build a gene content presence/absence matrix with R [i.e. rownames were set according to the first column (‘Gene’) and this first column was then deleted]. Then, for each pair of isolates, Pearson’s correlation coefficient was computed and tested by applying the function rcorr() (package Hmisc 5.1-3) to the presence/absence matrix. Tests were performed with 5059 degrees of freedom; the *P*-value was less than 2.2e-16. Correlation coefficients were displayed in a heatmap using ggplot2 (3.5.1).

### Antibiotic susceptibility testing

Susceptibility testing was performed by the disc diffusion method according to the guidelines of the Antibiogram Committee of the French Society for Microbiology (CA-SFM) (www.sfm-microbiologie.org). *Escherichia coli* strain ATCC 25922 and *Pseudomonas aeruginosa* ATCC 27853 were used for quality control. Susceptibility to 16 antibiotics (ticarcillin, ticarcillin-clavulanic acid, piperacillin, piperacillin-tazobactam, ceftazidime, cefepime, meropenem, imipenem, gentamicin, tobramycin, amikacin, streptomycin, ciprofloxacin and levofloxacin, minocycline, trimethoprim-sulfamethoxazole) were evaluated by disc diffusion on Muller-Hinton agar (BioRad). Results for piperacillin-tazobactam were confirmed using Sensititre plates (Thermofisher). Minimum inhibitory concentrations (MICs) of colistin, kanamycin, apramycin and tetracycline were determined by broth microdilution according to EUCAST recommendations.

## Results and discussion

### Investigating *A. baumannii* in a small animal ICU

To investigate the carriage of *A. baumannii* on pets in an ICU, 94 animals (60 dogs and 34 cats) were subjected to oral sampling over the course of a year with two main sampling periods in spring and autumn 2021 (April 2021 to 2022, Fig. 1). *A. baumannii* isolates were recovered from 12 animals, giving about 12.7% *A. baumannii* carriage (95%, confidence interval 5.9–19.6%). This prevalence is higher than but of the same order of magnitude as the observed carriage in veterinary non-ICU clinics (2.7–6.5% [14,31]). It is also close to the carriage observed in human hospitals (3.6–10.5%), although variations are observed between studies [32,34].

**Fig. 1.**
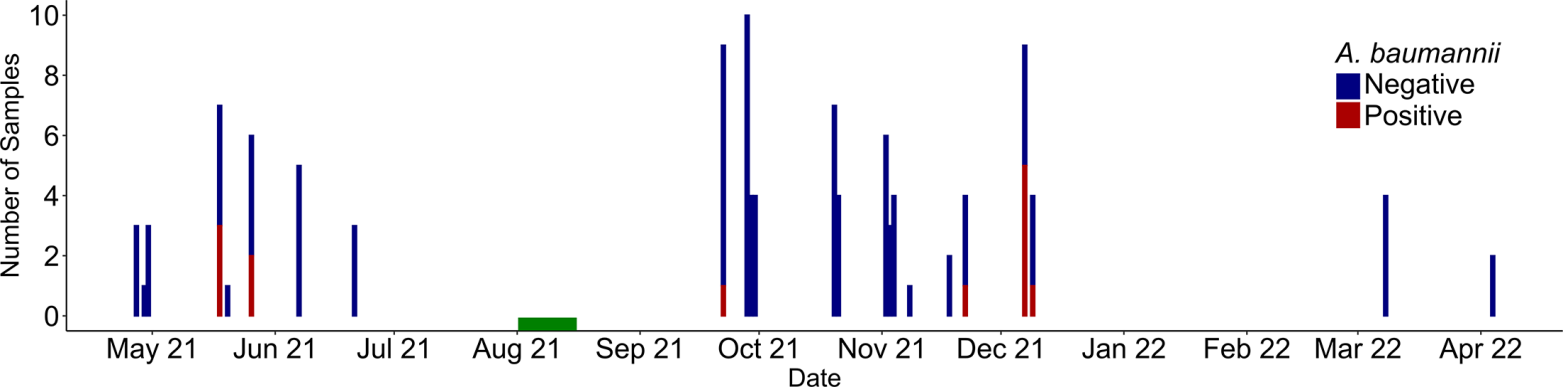
*A. baumannii* sampling timeline in a small animal ICU. The number of negative (blue) and positive (red) samples for *A. baumannii* are indicated during the sampling period. Summer cleaning and disinfection procedures are indicated by a green rectangle.

Over the entire sampling campaign, three animals were sampled twice during their hospitalization. Two of these, sampled in September and October 2021, 1 or 6 days apart, were not detected as contaminated. However, Dog#3, found negative on a first sampling, later tested positive. This suggests that it was contaminated during its 13 day stay in May 2021 (Table S1). Indeed, the duration of the hospitalization was identified as a risk factor of contamination (*P*<0.05, Fisher’s exact test). This latter correlation is consistent with human medicine observations [35] and suggested potential contamination originating from the ICU environment. The presence of *A. baumannii* was investigated within the ICU’s environment on two occasions (November 2021 and February 2022). Sampling of various fomites subjected to repeated handling (clinical table, surgical tray, door handles, computer keyboards, tape, shaver, infusion pump front board, ventilator front board, alcohol dispensers) did not reveal environmental contamination. However, *A. baumannii* was isolated from the keyboard of the lab computer in November 2021.

### Sporadic contamination with non-international clone strains

**Fig. 2.**
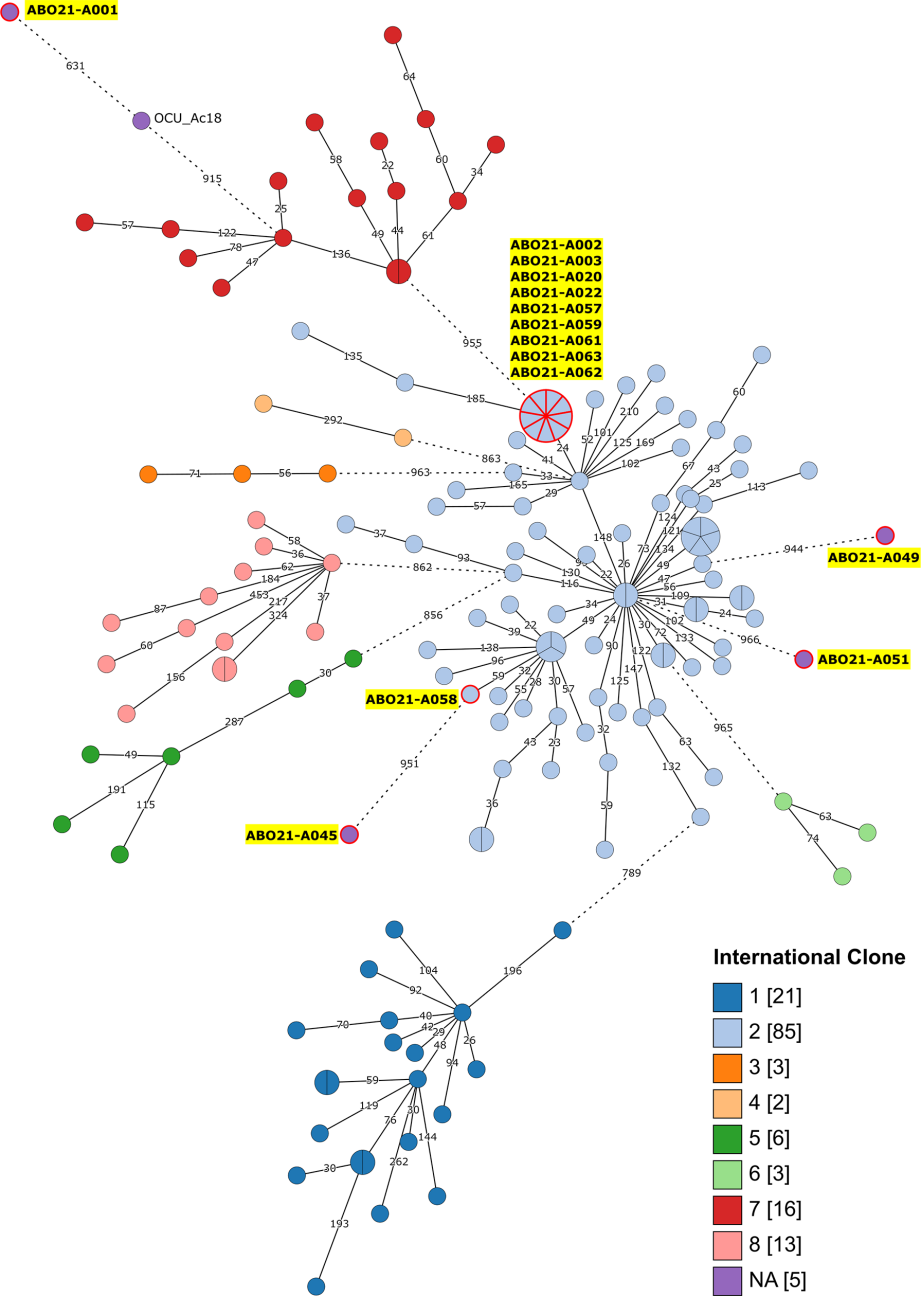
Minimum spanning tree of International Clone genomes and ICU isolates. Minimum spanning tree was built on the cgMLST (2390 genes) of 154 *A*. *baumannii* strains. International clones are displayed using different colours. Nodes differing by fewer than 20 alleles were collapsed together and branches longer than 500 alleles different are indicated with a hashed line. The 14 animal ICU isolates are indicated by yellow shadowed labels and red contour nodes. A list of strains and their metadata are available in Table S2. Strain names mentioned in the text are labelled. Number of allelic differences are given on each branch (log scale).

We sought to investigate the relationship between the various isolates by performing whole genome sequencing of the 13 *A*. *baumannii* strains collected from asymptomatic carriage and of the environment isolate. Complete genomes were obtained by hybrid assembly of short and long reads (see Methods). To place the study isolates in the *A. baumannii* phylogeny, and examine their relatedness with international clones as published by Mateo-Estrada [36], a cgMLST tree was reconstructed (Fig. 2 and Table S2). Minimum spanning tree analysis indicated that ten isolates belong to IC2 while four strains did not lie within known global clones. As those strains were associated with a single animal, they were considered as sporadic contamination.

The first non-global clone strain isolated, ABO21-A001, was assigned to the new ST^Pas^2532. This strain is sensitive to all the antibiotics tested, consistent with the absence of known antibiotic resistance genes in its genome. This ST^Pas^2532 is a single locus variant (*cpn60* gene) of ST^Pas^1639, rarely associated with human infection. Indeed, in the PubMLST database, only two strains isolated in Europe were assigned to ^Pas^ST1639 (one isolated in 2020 in France and one in Spain). To identify the closest genome to strain ABO21-A001, we used the Similar Genome Finder utility of WhatsGNU to query an *A. baumannii* genome database retrieved from the NCBI (4325 genomes obtained on 17 February 2023) [37]. WhatsGNU outputs ten genomes that have the highest numbers of exact protein allele matches to the reference genome. The closest match to ABO21-A001 shares only 44.4% of exact protein allele matches with a human-associated strain (OCUAc18, Japan, 2016), indicating a distant common ancestor (Fig. 2). Strain ABO21-A001 carries three small plasmids, two encoding a Rep3 family replication initiation protein (pABO21-A001-1 and -2) and one of the RepPriCT_1 group (pABO21-A001-3). Interestingly, the pABO21-A001-1 plasmid shows identity with pOCUAc18-2, one of the 11 plasmids hosted by its closest related strain OCUAc18 (Fig. S1A). Both plasmids carry a potential adaptive module with a set of putative amino acid-scavenging genes also described on plasmid pAbe229-15 carried by a clinical *Acinetobacter berezeniae* strain [38]. This suggests that these R3-T6 plasmids were acquired by a common ancestor of ABO21-A001 and OCUAc18. In contrast, other ABO21-A001 plasmids share identities with replicons found in ST1 strains isolated from human hosts, suggesting their more recent acquisition through horizontal gene transfer (Fig. S1A).

Similarly to strain ABO21-A001, we hypothesized that other non-international clones ABO21-A045 and ABO21-A051 represent contamination of the animal oral cavity by environmental *A. baumannii* strains. Indeed, these strains are both highly sensitive to antibiotics (Table S3) and lack closely related strains in the public database. This is consistent with a natural environmental origin [39]. Among the non-global clone strains, ST^Pas^578 strain ABO21-A049, collected on the lab computer keyboard, is singled out as multidrug resistant with resistance to β-lactams (ticarcillin, piperacillin), fluoroquinolones (ciprofloxacin, levofloxacin) and tetracycline (Table S3). The resistances are either chromosomal [*tetA*, *gyrA*(S84L)/*parC*(S81L)] or carried by plasmids. Indeed, two of the four plasmids carried by strain ABO21-A049 harbour resistance genes. pABO21-A049-2 has a new replication initiation protein and carries the *bla*_CARB-16_ gene and a transposase gene with 99.84% identity to sequences of a plasmid of the permafrost bacterium *Psychrobacter maritumus* (Fig. S1B) [40]. pABO21-A049-4 has an R3-T15 replication initiation protein and carries the *tet39* resistance gene.

### Contamination with an *A. baumannii* IC2 clone and potential zoonotic transmission

**Fig. 3.**
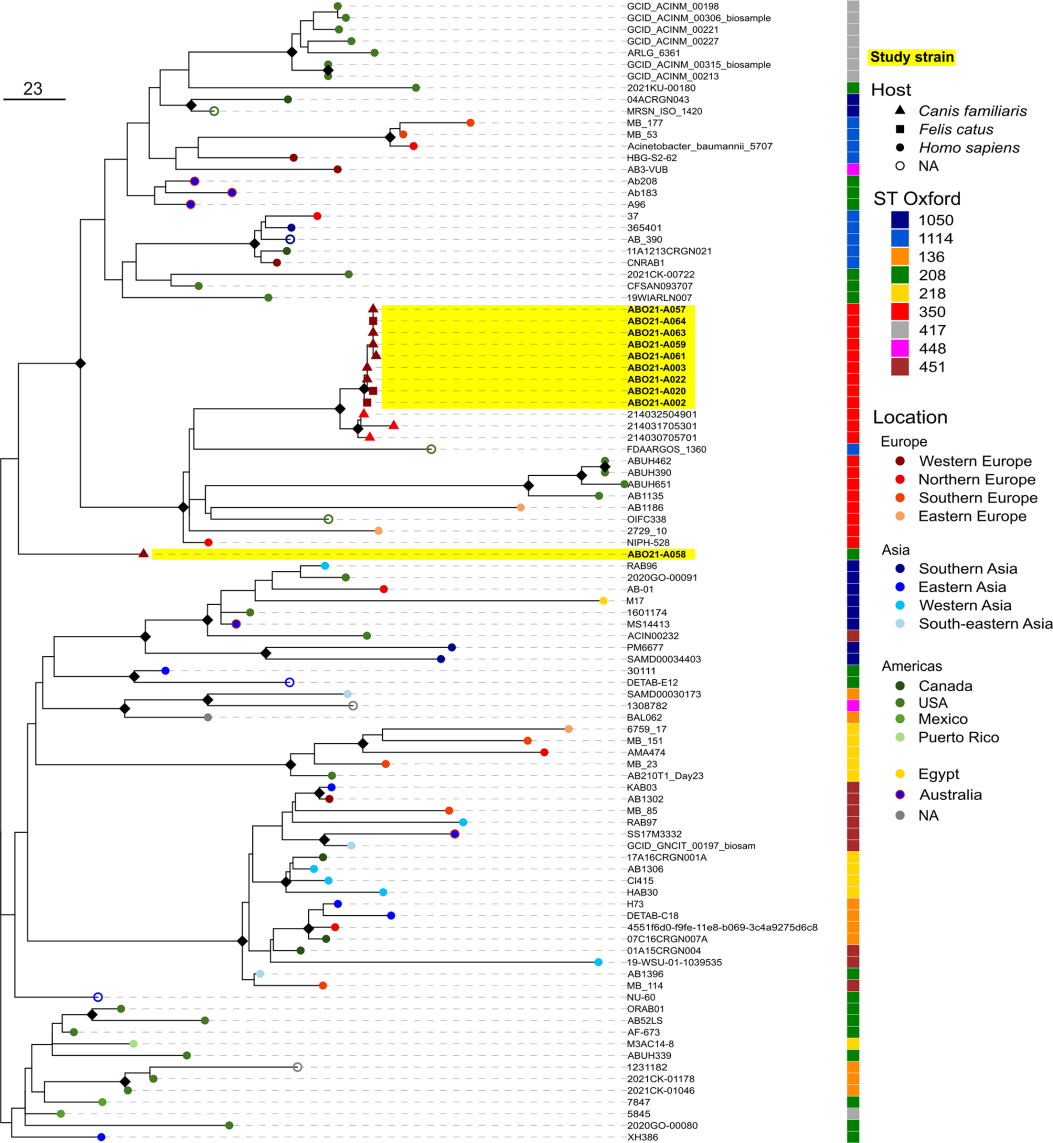
Phylogenetic proximity of animal ICU isolates with IC2 isolates. Maximum likelihood phylogenetic tree based on whole genome alignment without recombination of ten animal ICU isolates and 88 IC2 *A. baumannii* strains. ST Oxford is displayed in the coloured vector. Location is displayed as tree tip-coloured dots. Study strains are indicated by yellow shadowed labels. Host is displayed as tree tip shape. Bootstrap values equal to 100 are displayed as black diamonds. Bar, number of substitutions per genome. A list of strains and their metadata are available in Table S2.

Most strains (10/14) belong to the IC2 subclade ST^Pas^2 with nine monophyletic strains belonging to ST^Ox^350 and one to ST^Ox^208 (Fig. 2 and Table S1). We sought to locate more precisely these isolates among IC2 genomes through phylogenetic analysis including genomes selected using the Similar Genome Finder utility of the WhatsGNU program and the Pathogenwatch platform. ST^Ox^208 is one of the most predominant IC2 lineages worldwide and therefore is well represented in the genomic databases [41]. However, the ST^Ox^208 strain ABO21-A058 did not cluster with any strains even with isolates belonging to ST^Ox^208 (Fig. 3). The closest strains to ST^Ox^208 strain ABO21-A058 were Chinese human ST^Ox^208 isolate XH386, and Australian human ST^Ox^208 isolates (Ab183, Ab208 and A96) (Figs 3 and S2).

**Fig. 4.**
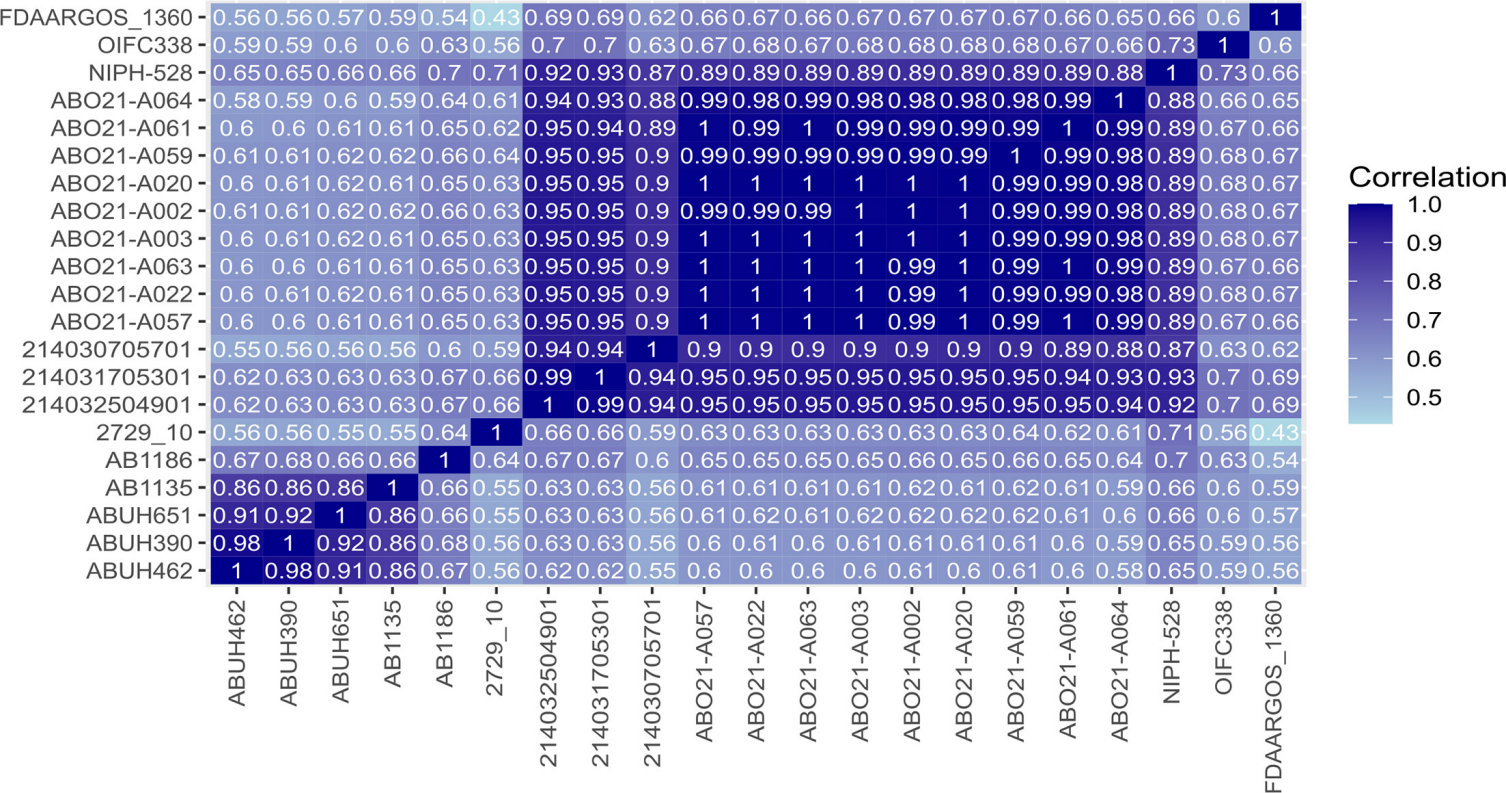
Correlation matrix of gene content. Heatmap displaying the Pearson correlation coefficient of gene content between pairs of selected ST^Ox^350 isolates.

By contrast, ST^Ox^350-ABO21 isolates cluster with three ST^Ox^350 strains from the Netherlands isolated in 2014 on diseased dogs during an outbreak in an animal ICU [42]. Whole genome sequence comparison identified only 25–26 SNPs between the early strain ABO21-A002 and the three 2014 Dutch dog strains. To further analyse their relationship, we conducted a gene content analysis, including genes experiencing recombination, and considering the ST350 strains of Fig. 3 including the distantly related Dutch human ST^Ox^350 isolate of 1982 (NIPH-528 [1]) (Fig. 4). With correlation coefficients between 0.88 and 0.95, these results underscore the high similarity between the animal strains from 2014 and 2021 from the Netherlands and France respectively. Patterns of clustering were consistent with the whole genome phylogeny (Fig. 3). These findings strongly favour the hypothesis of a country-to-country transmission of this clone mediated either by an animal or a human.

### Heteroresistance in clonal strains

All IC2 strains present a multidrug resistance profile but without resistance to tobramycin and cefepime nor to the last-line antibiotics colistin and carbapenems (Table S3). All ten IC2 strains showed resistance to fluoroquinolones, consistent with the genomic detection of the resistance alleles of the *gyrA* and *parC* genes leading to S81L and S84L substitutions respectively after translation of these topoisomerase IV genes. In the ST^Ox^208 ABO21-A058 strain, additional resistance to third-generation cephalosporine ceftazidime is due to an IS*Aba1* insertion upstream of the chromosomal *ampC* gene [43].

Accounting for their multidrug resistance profile, all IC2 strains also carry two IC2 large resistance islands, ABGRI1 and ABGRI2, commonly found in IC2 isolates. The ABO21-A058 strain carries an AbGRI1, typically inserted in the *comM* gene. This AbGRI1 variant is a version of ABGRI1-5 carried by the ST2 K16 strain deleted of IS*Aba1* and the *sul2* gene involved in sulphonamide resistance, deletion potentially due to recombination between *tniC* genes (Fig. S3). This ABGRI1 variant carries three resistance genes [*strA*, *strB* and tetA(B)*] accounting for resistance to streptomycin and tetracycline. Surprisingly, in this genomic context, *tetA(B*)* did not confer resistance to minocycline (Table S2), in contrast to previous observations [44]. This shorter version of ABGRI1 shares 100% nucleotide identity and coverage with ABGRI1 found in six closely related ST^Pas^2 strains: 5847, 7847, CNRAB1, BAL062, NU-60 and AB3-VUB (Fig. 3). The ABGRI1 from all ST^Ox^350-ABO21 strains are 100% identical and present an overall similar gene content with the ABGRI1 of strain ABO21-A058 but genes *tniC, tniA* and *sup* having allelic variations with those of ABO21-A058 (Fig. S3). The ABGRI1 from clonal ST^OX^350-ABO21 strains were identical to the Dutch 2014 outbreak strains in an animal ICU, showing their genetic proximity. By contrast, the GenBank non-redundant nucleotide database was queried but did not return any identical resistance island.

**Fig. 5.**
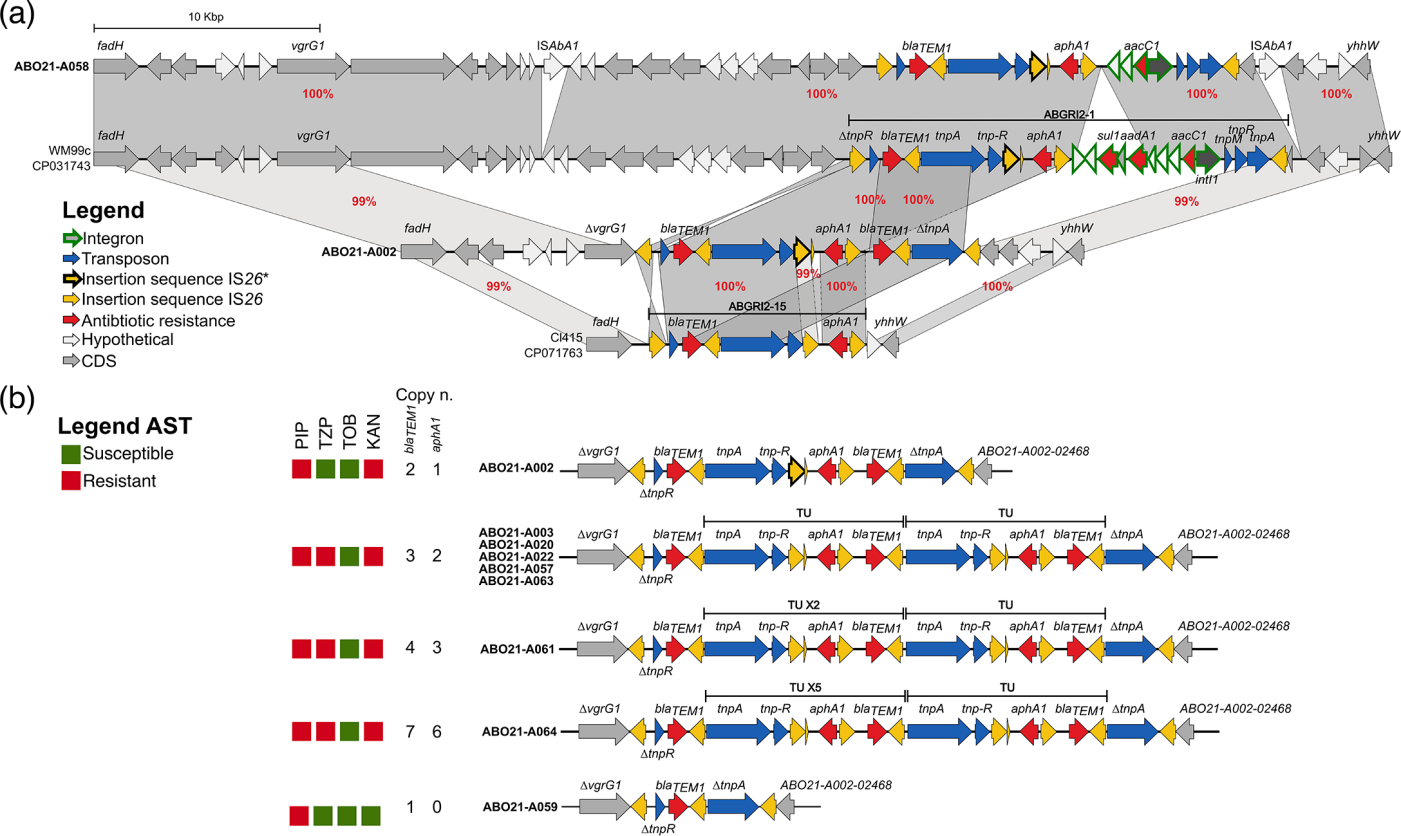
(**a**) Comparison of ABGRI2 islands found in IC2 strains isolated from an animal ICU in comparison to human isolates. Shades of grey indicate regions with 99 and 100% nucleotide identity. Horizontal lines represent the extent of ABGRI2-1 and 15 in the genomic context of strains WM99c and Cl415 respectively. (**b**) Genetic structures of ABGRI2 from ST^Ox^350-ABO21 strains. Putative translocatable units (TU) are indicated by a horizontal line. Number of TU repeats are noted (X2: two repeats, X5: five repeats) as the copy number of the *bla*_TEM1_ and *aphA1* genes. Levels of resistance to piperacillin (PIP), piperacillin-tazobactam (TZP), gentamicin (GEN), tobramycin (TOB) and kanamycin (KAN) were based on a disc diffusion assay or MICs when available and are indicated by coloured squares (resistant: red, susceptible: green). Legend to panel (a) also applies to panel (b).

All IC2 strains found in the animal ICU also carry variants of ABGRI2, IS*26-*flanked resistance islands, that are inserted in the same chromosomal location as previously described in IC2 strains isolated in Australia, the USA and Asia [45,47]. However, none of these ABGRI2 variants were found in any other strain in the GenBank database. ST^OX^208 ABO21-A058 carries an ABGRI2 that contains *bla*_TEM1_ and *aphA1* accounting for β-lactam and kanamycin resistance, respectively (Fig. 5a and Table S3). As in strains isolated from human infections, this genomic island is inserted at the same locus occupied by the ABGRI2-1 island in an IC2 WM99c strain isolated in 1999 [48]. Compared to this presumably earlier version, ABGRI2 of ABO21-A058 presents deletions of the class 1 integron resulting in the loss of two resistance genes (*sul1* and *aadA1*).

In comparison to the ABO21-A058 and WM99c isolates, the ABGRI2 in clonal ST^Ox^350-ABO21 strains exhibits a deletion in the DNA adjacent to ABGRI2, similarly to more recent IC2 isolates such as Cl415 (Fig. 5a). Furthermore, when compared to human isolates, they exhibit a duplication and deletion of a fragment that includes the *bla*_TEM1_ gene and the gene encoding the TnpA transposase (Fig. 5a). Additionally, there is an inversion of the IS*26* at the left end of their islands (Fig. 5b). These islands harbour multiple direct repeats of IS*26*, which may explain the expansion of resistance genes observed in most ST^OX^350-ABO21 isolates (Fig. 5b). Similar to the WM99c strain, the ABGRI2 in the early isolate ABO21-A002 also harbours one copy of IS26*, a variant of IS*26* that has been demonstrated to exhibit enhanced transposition activity [49]. IS26* was replaced by IS*26* in subsequent isolates (Fig. 5b).

In *E. coli* and *A. baumannii*, direct repeats of IS*26* have been demonstrated to mediate increasing copy numbers of adjacent antibiotic resistance genes and to extend their initial spectrum of resistance [50,52]. For both bacterial species, direct repeats of IS*26* flanking resistance genes allow amplification of a translocatable unit (TU) and thereby resistance gene amplification. TEM-1 β-lactamase is usually inhibited by tazobactam, but a higher copy number of the *bla*_TEM1_ gene gives a sufficient level of BlaTEM-1 enzyme to overcome tazobactam inactivation resulting in increased resistance to piperacillin-tazobactam [50]. IS*26*-mediated resistance gene amplification was also observed in *A. baumannii* with amplification of the *aphA1* resistance gene [51]. This latter gene usually confers resistance to kanamycin and neomycin but 11 *aphA1* copies conferred resistance to tobramycin [51,52].

Similarly, direct repeats of IS*26* in the ABGRI2 of ST^Ox^350-ABO21 strains may have generated translocable units (TUs) encompassing the *bla*_TEM1_ and *aphA1* genes (Fig. 5b). The varying number of repeats of the TU, observed across strains, leads to heterogeneous resistance profiles to piperacillin-tazobactam (Fig. 5b). Three (strains ABO21-A003, 020, 022, 057, 063), four (ABO21-A061) and seven (ABO21-A064) copies of the *bla*_TEM1_ gene conferred resistance to the piperacillin-tazobactam association as assayed under laboratory conditions. However, the six copies of the *aphA1* gene in strain ABO21-A064 may not be sufficient to produce tobramycin resistance.

By contrast, strain ABO21-A059 exhibits susceptibility to piperacillin-tazobactam and kanamycin (Fig. 5b). This loss of resistance in this latter isolate is attributable to a deletion event mediated by recombination between two IS*26*. Interestingly, strain ABO21-A059 was isolated on the same day, but on different animals, as strains ABO21-A057 and ABO21-A063, all presenting varying degrees of resistance. This underlines the role of gene amplification as a main mechanism of heteroresistance between isolates [53]. Of note, the ABO21-A064-carrying animal (Cat#10) received high-dose treatment with a penicillin – β-lactamase inhibitor combination the day before sampling in comparison to other sampled animals. As overexpression of TEM-1 also confers resistance to ampicillin-sulbactam in *A. baumannii*, we hypothesize that this treatment may have selected *bla*_TEM1_ gene amplification [54]. Ampicillin-sulbactam combination is the most prescribed antibiotic in this animal ICU whereas piperacillin-tazobactam combination is restricted to human medicine. Our results indicate that, in the case of gene amplification-mediated heteroresistance, use of ampicillin-sulbactam could promote cross-resistance to piperacillin-tazobactam.

### Transmission within the animal ICU

**Fig. 6.**
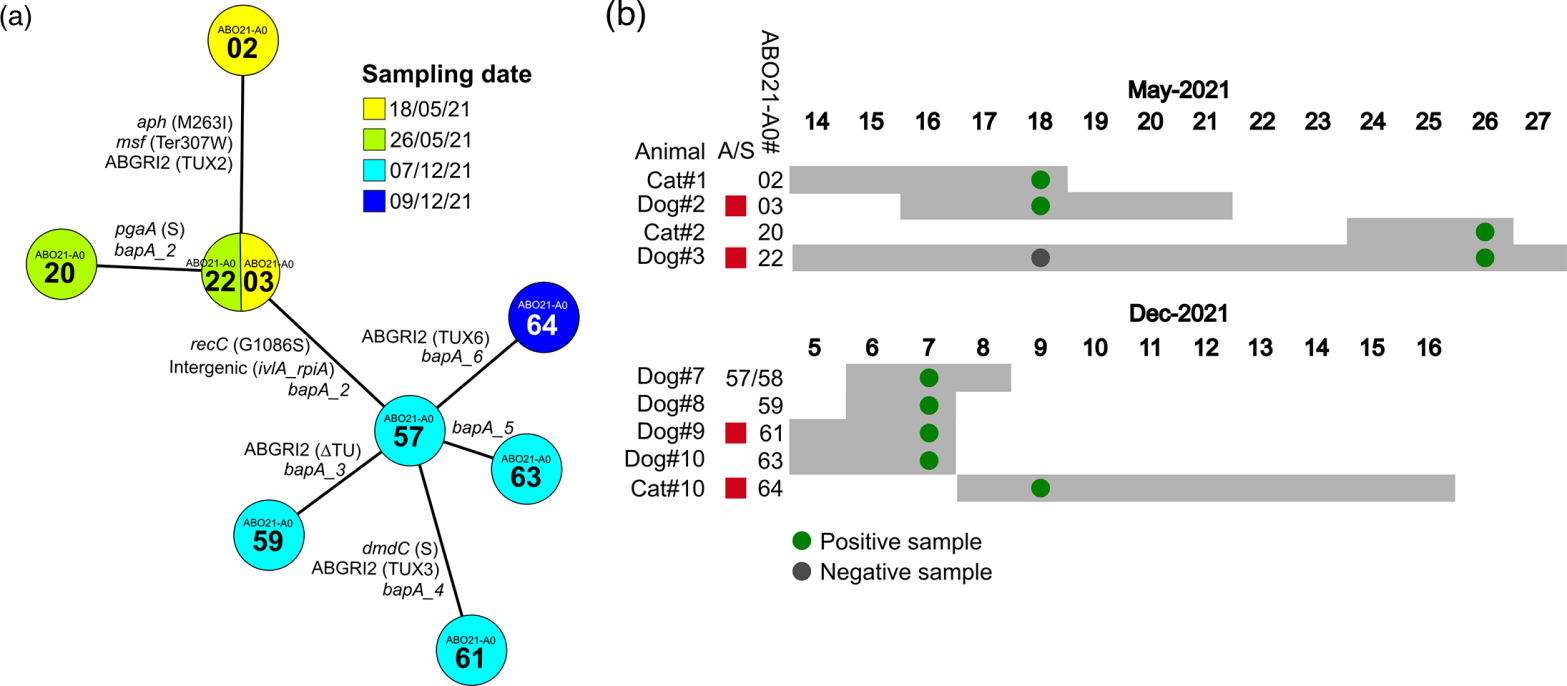
(**a**) Minimum spanning tree depicting the genetic relationships among ST^Ox^350 isolates based on all genome variations. The connecting line lengths are proportional to the numbers of genetic variations. For protein-encoding genes, the effects induced by SNPs are indicated either by S standing for a synonymous SNP or by the effect on the gene product when non-synonymous. Sampling dates are colour coded. (**b**) Timeline of ST^Pas^2 strain isolation during an animal’s hospital stay (grey rectangles). Day of positive samples is indicated by a green dot. Dog #3 was sampled twice with one negative sample (grey dot). Animals that received ampicillin-sulbactam (A/S) treatment are indicated by red squares.

SNP analysis between ST^Ox^350 strains revealed limited allelic variation between isolates with at most five SNPs between the early strain ABO21-A002 and the late strain ABO21-A061 (Fig. 6). Two non-synonymous SNPs in the *aph* and *msf* genes distinguish the early strain ABO21-A002 from other strains. As the *aph* gene sequence is identical in genomes from closely related human ST^Ox^350 isolates, such as strain NIPH-528 (Fig. 3), we speculated that a mutation arose and was maintained in later isolates. By contrast, the gene encoding a putative transporter (*msf*) of later clones is identical to that found in closely related human ST^Ox^350 isolates, indicating that this mutation arose in ABO21-A002 from the founder genotype (*in extenso* the genotype of closely related human ST^Ox^350 isolates). Indeed, the relatedness of ABO21-A002’s ABGRI2 with islands of other IC2 strains, such as the presence of IS26*, suggests that this strain derived from the founder genotype (Fig. 5a). Lastly, the isolates present insertion or deletion events within the *bapA* gene (Fig. S4). The latter encodes a large secreted protein, BapA, involved in biofilm formation and adhesion [55]. The *bapA* gene is composed of repeated modules that may be subjected to rearrangement leading to six variants in the ST^Ox^208 strains [56].

The allelic distances observed are consistent with the sampling dates, revealing clustering for strains isolated in May 2021 and those isolated in December 2021.

Altogether, comparison of clonal strain genomes revealed that over a 7 month period at most five isolated SNPs occurred. This apparent slow evolutionary rate is consistent with the evolutionary rate of ~10 SNPs per year between hosts suggested for human IC2 clones and indicate survival in the environment [57,58]. Noteworthy, Dog#2 received ampicillin/sulbactam treatments that may have selected an ABO21-A003 strain bearing *bla*_TEM1_ amplification (Figs5b  6). Genomes of ABO21-A003 and A022 strains are identical but sampled 8 days apart. This indicates that ABO21-A003 may then have been transmitted from Dog#2 to Dog#3 or from a common source (Fig. 6b). The lengthy stay of Dog#3 may have allowed the persistence and subsequent transmission of ABO21-A022 strain to Cat#2. In addition, strains isolated in December 2021 all exhibit a substitution in the *recC* gene (Fig. 6a). This gene encodes a component of the RecBCD heterotrimeric enzyme involved in the repair of dsDNA breaks [59]. Of note, a previous study on *A. baumannii* strains isolated from human infections also identified SNPs in the *recJ* gene involved in DNA repair and replication, indicating a potential role of these genes in mobile genetic element-mediated evolution.

Although ST^Ox^350 strains were repeatedly isolated from hospitalized animals, this was not the case for the other IC2 strain of ST^Ox^208 (ABO21-A058) isolated on a single animal (Dog#7). Interestingly, Dog#7 was sampled on 7 December, and found to be contaminated with the ST^OX^208 strain (ABO21-A058) but also with an ST^Ox^350 strain (ABO21-A057). Dog#7’s medical record reported that it was admitted to the ICU due to a lip bite (Table S1). This medical condition suggests extensive manipulation of the mouth, either by its owners prior to admission, or during hospitalization. Of note, all sampling was performed by oral swabbing of the animals. Contamination with the ST^Ox^208 strain, a more widely distributed human ST, may therefore have originated externally to the ICU.

Altogether, these results suggest that the parental strain of ST^Ox^350 strains was introduced in the ICU, either by Cat#1 or by a member of the medical staff. Subsequent transmission to other hospitalized animals was most probably indirect through contaminated medical devices. Indeed, the slow nucleotide evolution rate favours the hypothesis of contamination of an unidentified specific device used 7 months apart in the ICU. This result strongly suggests common mechanisms of these veterinary and human IC2 isolates persisting on abiotic surfaces regardless of disinfection procedures, and to which resistance can be mediated by multiple efflux pumps conserved in *A. baumannii* genomes [46,48].

## Concluding remarks

Full genome sequencing and hybrid assemblies allowed us to trace the origin of *A. baumannii* strains contaminating a companion animals hospital ward. Our results confirm that human activities probably spread IC2 strains over a large geographical area. However, they highlight a surprising specificity of an IC2 clade to animal or animal care settings that requires further investigation.

## supplementary material

10.1099/mgen.0.001292 Uncited Supplementary Material 1.

## Abbreviations

cgMLST: core gene multilocus sequence typing
IC: International Clone
ICU: intensive care unit
MIC: minimum inhibitory concentration
TU: translocatable unit
