# Supplementary material for: Genomics unveils country-to-country transmission between animal hospitals of a multidrug-resistant and sequence type 2 Acinetobacter baumannii clone
Source: Microb Genom. 2024 Oct 14;10(10):001292. doi: 10.1099/mgen.0.001292 (PMC11472879; doi:10.1099/mgen.0.001292)
Supplement: Uncited Supplementary Material 1. [file mgen-10-01292-s001.pdf]

Genomics unveils country-to-country transmission between animal hospitals of a multidrug resistant and Sequence Type 2 *Acinetobacter baumannii* clone

Amédée André[#], Julie Plantade[#], Isabelle Durieux, Pauline Durieu, Anne-Sophie Godeux, Maxence Decellieres, Céline Pouzot-Nevoret, Samuel Venner, Xavier Charpentier[◊] and, Maria-Halima Laaberki[◊,*].

# equal contributions (co-first authors, alphabetical order)

◊ equal contributions (senior authorship, alphabetical order)

* Correspondence to Maria-Halima Laaberki, maria-halima.laaberki@vetagro-sup.fr

Supplementary material

Table S1. Study strains and genome information

Table S2. List of strains used for phylogeny analysis.

Table S3. Antibiotic susceptibility testing

Figure S1. Structures of plasmids found in $ST^{Pas}2532$ strain (ABO21-A001) and $ST^{Pas}578$ strain (ABO21-A049)

Figure S2. Phylogenetic proximity of animal ICU isolates with IC2 isolates

Figure S3. Structures of ABGRI1 resistance islands found in GC2 strains isolated from animal ICU in comparison to human isolate K16.

Figure S4. Comparative alignment of the *bapA* gene and its surrounding region in eight $ST^{OX}350$ isolates with the early ABO21-A002 isolate.

Table S1. Study strains and genome information (numeric ordering)

Strain name	Origin	Sampling date	Pathology at admission	ST^{Pas}	ST^{Ox}	Chromosome size in pb (Accession number)	Plasmid size in pb (Accession number)
ABO21-A001	Dog#1	18/05/2021	Pericardial, abdominal and pleural effusion	2532	New	3997947 (CP136183)	14128 (CP136184) , 6479 (CP136185), 5345 (CP136186)
ABO21-A002	Cat#1	18/05/2021	Congestive heart failure	2	350	3831295 (CP136182)	
ABO21-A003	Dog#2	18/05/2021	Gastric Dilation and Volvulus	2	350	3841318 (CP136181)	
ABO21-A020	Cat#2	26/05/2021	Squamous cell carcinoma of the trachea	2	350	3840805 (CP136180)	
ABO21-A022	Dog#3	26/05/2021	Acute Kidney Injury	2	350	3841316 (CP136179)	
ABO21-A045	Dog#4	22/09/2021	Neurophathy	1384	New	3962234 (CP136178)	
ABO21-A049	Env.	08/11/2021	Not applicable	578	New	3915620 (CP136173)	9474 (CP136174), 8066 (CP136175), 7813 (CP136176), 6010 (CP136177)
ABO21-A051	Dog#5	18/11/2021	Discospondylitis	2247	New	3945407 (CP136172)	
ABO21-A057	Dog#7	07/12/2021	Lip bite	2	350	3842503 (CP136171)	
ABO21-A058				2	208	3877162 (CP136169)	4804 (CP136170)
ABO21-A059	Dog#8	07/12/2021	Convulsive seizure associated with diabetes	2	350	3824214 (CP136168)	
ABO21-A061	Dog#9	07/12/2021	Gastric Dilation and Volvulus	2	350	3850979 (CP136167)	
ABO21-A063	Dog#10	07/12/2021	Acute Kidney Injury	2	350	3842192 (CP136166)	
ABO21-A064	Cat#3	09/12/2021	Pyothorax	2	350	3877839 (CP136165)	

Table S2. List of strains used for genomic analysis.

Associated with Figure 2.

Strain	RefSeq	Host	Geographic_location	Isolation_date	IC	Pasteur	Oxford
ABO21-A001	GCF_033192035.1	Canis familiaris	France	18/05/2021	NA	2532	New
ABO21-A002	GCF_033191435.1	Catus felis	France	18/05/2021	2	2	350
ABO21-A003	GCF_033191195.1	Canis familiaris	France	18/05/2021	2	2	350
ABO21-A020	GCF_033190815.1	Catus felis	France	26/05/2021	2	2	350
ABO21-A022	GCF_033190675.1	Canis familiaris	France	26/05/2021	2	2	350
ABO21-A045	GCF_033190095.1	Canis familiaris	France	22/09/2021	NA	1384	New
ABO21-A049	GCF_033469325.1	Catus felis	France	08/11/2021	NA	578	New
ABO21-A051	GCF_033190015.1	Canis familiaris	France	18/11/2021	NA	2247	New
ABO21-A057	GCF_033189995.1	Canis familiaris	France	07/12/2021	2	2	350
ABO21-A058	GCF_033189955.1	Canis familiaris	France	07/12/2021	2	2	208
ABO21-A059	GCF_033189435.1	Canis familiaris	France	07/12/2021	2	2	350
ABO21-A061	GCF_033188675.1	Canis familiaris	France	07/12/2021	2	2	350
ABO21-A063	GCF_033187975.1	Canis familiaris	France	07/12/2021	2	2	350
ABO21-A064	GCF_033187155.1	Catus felis	France	09/12/2021	2	2	350
OCU_Ac18	GCF_019703285.1	Homo_sapiens	Japan	2016	NA	NA	2736
CI415	GCF_018604365.1	Homo sapiens	Lebanon	2017	2	2	218
KAB03	GCF_001806385.1	Homo sapiens	South_Korea	2015	2	2	451
KAB01	GCF_001806345.1	Homo sapiens	South_Korea	2015	2	2	451
XH859	GCF_001573085.1	Homo sapiens	China	2010	2	2	368
XH386	GCF_001026965.1	Homo sapiens	China	2017	2	2	208
IHSS3526	GCF_002927895.1	Homo_sapiens	Honduras	2016	1	1	1590
AB4991	GCF_001612415.1	Homo_sapiens	USA	2008	1	1	207
A388	GCF_001512055.1	Homo_sapiens	Greece	2002	1	1	439
D81	GCF_001511995.1	Homo_sapiens	Australia	2010	1	1	441
R1B	GCF_000623035.1	Homo_sapiens	Saudi_Arabia	2011	1	1	441
RBH3	GCF_001512075.1	Homo_sapiens	Australia	2002	1	1	781
6772166	GCF_001512175.1	Homo_sapiens	Australia	2002	1	1	781
AB4448	GCF_001612255.1	Homo_sapiens	USA	2007	1	1	945
AB5674	GCF_001612435.1	Homo_sapiens	USA	2009	1	1	945
D3208	GCF_001512255.1	Homo_sapiens	Australia	1997	1	1	231
A297	GCF_001512215.1	Homo_sapiens	Netherlands	1984	1	1	231
NIPH_290	GCF_000369325.1	Homo_sapiens	Czech_Republic	1994	1	1	231
AB5197	GCF_001612455.1	Homo_sapiens	USA	2008	1	1	231
MEX11594	GCF_001549575.1	Homo_sapiens	Mexico	2011	1	1	231
IHIT25424	GCF_001704695.1	Homo_sapiens	Germany	2011	1	1	231
13	GCF_002245595.1	Homo_sapiens	Brazil	2012	1	1	231

**237**	GCF_003670075.1	Homo_sapiens	Togo	2016	1	1	231
**AB3340**	GCF_001612005.1	Homo_sapiens	USA	2006	1	20	449
**71**	GCF_002119265.1	Homo_sapiens	Spain	2010	1	81	1868
**AB3927**	GCF_001612115.1	Homo_sapiens	USA	2007	1	717	441
**ZQ3**	GCF_002838025.2	Homo_sapiens	Iraq	2016	1	717	498
**ZQ1**	GCF_002803655.2	Homo_sapiens	Iraq	2016	2	2	1052
**O1-3**	GCF_007113795.1	Homo_sapiens	China	2018	2	2	136
**IHIT29106**	GCF_001704685.1	Homo_sapiens	Germany	2014	2	2	1421
**4300STDY7045777**	GCF_900495555.1	Homo_sapiens	Thailand	2016	2	2	1423
**2004BJAB10**	GCF_000804655.1	Homo_sapiens	China	2004	2	2	1513
**4300STDY7045764**	GCF_900495335.1	Homo_sapiens	Thailand	2016	2	2	1634
**RCS4**	GCF_006369835.1	Homo_sapiens	France	2017	2	2	1634
**4300STDY7045703**	GCF_900494845.1	Homo_sapiens	Thailand	2016	2	2	1933
**A186**	GCF_001420505.1	Homo_sapiens	South_Africa	NA	2	2	204
**14A771**	GCF_004136905.1	Homo_sapiens	France	2014	2	2	348
**15A831**	GCF_004136925.1	Homo_sapiens	France	2015	2	2	348
**4300STDY7045768**	GCF_900495485.1	Homo_sapiens	Thailand	2016	2	2	349
**NIPH_528**	GCF_000369285.1	Homo_sapiens	Netherlands	1982	2	2	350
**1999BJAB11**	GCF_000805035.1	Homo_sapiens	China	1999	2	2	350
**Ab_Crete**	GCF_001420515.1	Homo_sapiens	United Kingdom	2011	2	2	350
**Ab158_GEIH-2000**	GCF_001950065.1	Homo_sapiens	Spain	2000	2	2	350
**6009-2**	GCF_003052285.1	Homo_sapiens	Portugal	2010	2	2	350
**ABAY11001**	GCF_009736935.1	Homo_sapiens	South Korea	2011	2	2	357
**2011BJAB7**	GCF_000804645.1	Homo_sapiens	China	2011	2	2	369
**ABAY13010**	GCF_009736485.1	Homo_sapiens	South Korea	2013	2	2	369
**4300STDY7045691**	GCF_900494665.1	Homo_sapiens	Thailand	2016	2	2	436
**KT_2015_40**	GCF_009760575.1	Homo_sapiens	Greece	2015	2	2	436
**AB4932**	GCF_001612365.1	Homo_sapiens	USA	2008	2	2	452
**MDRAB16**	GCF_003352595.1	Homo_sapiens	Taiwan	2010	2	2	455
**KSPU17**	GCF_003352525.1	Homo_sapiens	Taiwan	2013	2	2	455
**4300STDY7045862**	GCF_900496445.1	Homo_sapiens	Thailand	2016	2	2	493
**AB10**	GCF_003007215.1	Homo_sapiens	China	2013	2	2	540
**MDRAB41**	GCF_003352615.1	Homo_sapiens	Taiwan	2009	2	2	544
**MDRAB58**	GCF_003352675.1	Homo_sapiens	Taiwan	2009	2	2	545
**XH515**	GCF_001863465.1	Homo_sapiens	China	2014	2	2	547
**AB4_2015**	GCF_003007275.1	Homo_sapiens	China	2015	2	2	548
**NIPH_24**	GCF_000367885.1	Homo_sapiens	Czech_Republic	1991	2	2	556
**Ab_12**	GCF_002238075.1	Homo_sapiens	Ivory_Coast	2013	2	2	556
**2011ZJAB1**	GCF_000804895.1	Homo_sapiens	China	2011	2	2	643
**ZJ06-200P5-1**	GCF_002093815.1	Homo_sapiens	China	2012	2	2	643
**ABAY13015**	GCF_009736405.1	Homo_sapiens	South_Korea	2013	2	2	858

**4300STDY7045869**	GCF_900496565.1	Homo_sapiens	Thailand	2016	2	2	938
**XH508**	GCF_001863345.1	Homo_sapiens	China	2014	2	2	191
**ABAY09003**	GCF_009737925.1	Homo_sapiens	South_Korea	2009	2	2	191
**2011BJAB3**	GCF_000804635.1	Homo_sapiens	China	2011	2	2	218
**M3AC14-8**	GCF_001672535.1	Homo_sapiens	Puerto_Rico	2014	2	2	218
**KMDRAB15**	GCF_003352495.1	Homo_sapiens	Taiwan	2012	2	2	218
**AbMDR-GLH5**	GCF_001541525.1	Homo_sapiens	Spain	2011	2	2	218
**RCS2**	GCF_006369755.1	Homo_sapiens	France	2015	2	2	218
**2011BJAB1**	GCF_000805295.1	Homo_sapiens	China	2011	2	2	368
**ABAY09002**	GCF_009737985.1	Homo_sapiens	South_Korea	2009	2	2	368
**s228**	GCF_003356575.1	Homo_sapiens	China	2012	2	2	381
**AK_2015_20**	GCF_009760775.1	Homo_sapiens	Greece	2015	2	2	425
**TR_2016_34**	GCF_009760535.1	Homo_sapiens	Greece	2016	2	2	425
**AB4_2011**	GCF_003335955.1	Homo_sapiens	HongKong	2011	2	2	457
**2015ZJAB3**	GCF_001617835.1	Homo_sapiens	China	2014	2	2	784
**XH722**	GCF_001862265.1	Homo_sapiens	China	2013	2	2	784
**Ab77**	GCF_900117945.2	Homo_sapiens	Australia	1999	2	2	208
**NIPH_2061**	GCF_000368225.1	Homo_sapiens	Czech Republic	2003	2	2	208
**BIDMC_56**	GCF_000692095.1	Homo_sapiens	USA	2013	2	2	208
**BJ7**	GCF_000787335.1	Homo_sapiens	China	2012	2	2	208
**ISAB51**	GCF_003352485.1	Homo_sapiens	Taiwan	2010	2	2	208
**AB067**	GCF_001909135.1	Homo_sapiens	India	2013	2	2	195
**GML-KP7-Col_R_AB-TR**	GCF_003183965.1	Homo_sapiens	Turkey	2017	2	2	195
**GE_2017_62**	GCF_009759995.1	Homo_sapiens	Greece	2017	2	2	195
**NIPH_1362**	GCF_000368125.1	Homo_sapiens	Czech Republic	2000	2	47	437
**AbMDR-GLH1**	GCF_001541875.1	Homo_sapiens	Spain	2011	2	187	208
**AbMDR-GLH2**	GCF_001541455.1	Homo_sapiens	Spain	2011	2	745	208
**4300STDY7045742**	GCF_900495225.1	Homo_sapiens	Thailand	2016	2	823	457
**XH733**	GCF_001862385.1	Homo_sapiens	China	2013	2	880	208
**XH685**	GCF_001864615.1	Homo_sapiens	China	2014	2	2	451
**S18**	GCF_002950525.1	Homo_sapiens	USA	2011	2	2	451
**ABAY13014**	GCF_009736395.1	Homo_sapiens	South_Korea	2013	2	2	451
**RCS1**	GCF_006369735.1	Homo_sapiens	France	2014	2	2	451
**SI_2017_69**	GCF_009759905.1	Homo_sapiens	Greece	2017	2	2	451
**AB3560**	GCF_001612015.1	Homo_sapiens	USA	2006	3	3	928
**AB4456**	GCF_001612215.1	Homo_sapiens	USA	2007	3	3	928
**NIPH_1669**	GCF_000368105.1	Homo_sapiens	Netherlands	1997	3	3	106
**MC75**	GCF_003583665.1	Homo_sapiens	Bolivia	2016	4	15	236
**NIPH_1734**	GCF_000368245.1	Homo_sapiens	Czech_Republic	2001	4	15	950
**CU032113**	GCF_001700445.1	Homo_sapiens	USA	2013	5	79	124
**MC17**	GCF_003596215.1	Homo_sapiens	Bolivia	2016	5	79	233

**MC38**	GCF_003595875.1	Homo_sapiens	Bolivia	2016	5	79	233
**MCR6056**	GCF_002928135.1	Homo_sapiens	Honduras	2015	5	156	758
**A158**	GCF_004209215.1	Homo_sapiens	Mexico	2012	5	156	758
**Ab5038**	GCF_004794205.1	Homo_sapiens	Mexico	NA	5	156	758
**AB4332**	GCF_004282835.1	Homo_sapiens	Brazil	2016	6	78	944
**830**	GCF_009445385.1	Homo_sapiens	Georgia	2018	6	78	1104
**3365**	GCF_009445395.1	Homo_sapiens	Georgia	2018	6	78	944
**MC31**	GCF_003595955.1	Homo_sapiens	Bolivia	2016	7	25	1489
**MC32**	GCF_003595935.1	Homo_sapiens	Bolivia	2016	7	25	1489
**MC63**	GCF_003595685.1	Homo_sapiens	Bolivia	2016	7	25	1519
**MC64**	GCF_003595675.1	Homo_sapiens	Bolivia	2016	7	25	1519
**MC71**	GCF_003584455.1	Homo_sapiens	Bolivia	2016	7	25	1528
**MC77**	GCF_003584375.1	Homo_sapiens	Bolivia	2016	7	25	1528
**MC39**	GCF_003595865.1	Homo_sapiens	Bolivia	2016	7	25	1529
**MC51**	GCF_003595815.1	Homo_sapiens	Bolivia	2016	7	25	1529
**HEU3**	GCF_002927855.1	Homo_sapiens	Honduras	2016	7	25	1588
**AMA42**	GCF_009645465.1	Homo_sapiens	NA	2017	7	25	690
**AB2828**	GCF_001611995.1	Homo_sapiens	USA	2006	7	25	993
**AB3638**	GCF_001612095.1	Homo_sapiens	USA	2007	7	25	993
**MCR10179**	GCF_002927965.1	Homo_sapiens	Honduras	2015	7	25	229
**AMA3**	GCF_009645745.1	Homo_sapiens	NA	2015	7	25	229
**MC1**	GCF_003597385.1	Homo_sapiens	Bolivia	2015	7	991	1518
**MC53**	GCF_003595805.1	Homo_sapiens	Bolivia	2016	7	991	1518
**4300STDY7045695**	GCF_900494795.1	Homo_sapiens	Thailand	2016	8	10	447
**NIPH_335**	GCF_000369205.1	Homo_sapiens	Czech_Republic	1994	8	10	447
**LAC-4**	GCF_000623015.1	Homo_sapiens	USA	1997	8	10	447
**2011ZJAB4**	GCF_000805215.1	Homo_sapiens	China	2011	8	10	447
**MCR9238**	GCF_002927775.1	Homo_sapiens	Honduras	2015	8	10	447
**ABAY14008**	GCF_009736145.1	Homo_sapiens	South_Korea	2014	8	10	447
**TVGH-612**	GCF_008123485.1	Homo_sapiens	Taiwan	2002	8	10	447
**4300STDY7045774**	GCF_900495565.1	Homo_sapiens	Thailand	2016	8	10	585
**T214**	GCF_000802915.1	Homo_sapiens	Thailand	2010	8	10	585
**2004ZJAB5**	GCF_000804955.1	Homo_sapiens	China	2004	8	23	642
**XH683**	GCF_001864555.1	Homo_sapiens	China	2012	8	23	642
**ZQ9**	GCF_002837885.2	Homo_sapiens	Iraq	2016	8	575	582
**ZQ10**	GCF_002837905.2	Homo_sapiens	Iraq	2016	8	575	582

Associated with Figure 3

biosample	strain	location	year	host	ST_Pasteur	ST_Oxford
**SAMN37643086**	ABO21-A002	France	2021	*Felis catus*	2	350
**SAMN37654775**	ABO21-A003	France	2021	*Canis familiaris*	2	350
**SAMN37654776**	ABO21-A020	France	2021	*Felis catus*	2	350
**SAMN37654777**	ABO21-A022	France	2021	*Canis familiaris*	2	350
**SAMN37654781**	ABO21-A057	France	2021	*Canis familiaris*	2	350
**SAMN37654782**	ABO21-A058	France	2021	*Canis familiaris*	2	1806, 208
**SAMN37654783**	ABO21-A059	France	2021	*Canis familiaris*	2	350
**SAMN37654784**	ABO21-A061	France	2021	*Canis familiaris*	2	350
**SAMN37654785**	ABO21-A063	France	2021	*Canis familiaris*	2	350
**SAMN37654786**	ABO21-A064	France	2021	*Felis catus*	2	350
**SAMD00034403**	SAMD00034403	Nepal	2013	*Homo sapiens*	2	1050
**SAMEA9273376**	PM6677	India	2019	*Homo sapiens*	2	1050
**SAMEA9459702**	AB-01	Netherlands	2019	*Homo sapiens*	2	1050
**SAMN04480654**	MRSN_ISO_1420	USA	2010	NA	2	1050
**SAMN07258622**	04ACRGN043	Canada	2010	*Homo sapiens*	2	1050
**SAMN17266007**	M17	Egypt	2020	*Homo sapiens*	2	1050
**SAMN17767272**	2020GO-00091	USA	2020	*Homo sapiens*	2	1050
**SAMN21392324**	1601174	USA	2015	*Homo sapiens*	2	1050
**SAMN27010290**	RAB96	Saudi Arabia	2019	*Homo sapiens*	2	1050
**SAMN14859482**	MS14413	Australia	2016	*Homo sapiens*	2	1050, 2058
**SAMEA10096945**	365401	Afganistan	NA	*Homo sapiens*	2	1114
**SAMEA2241521**	37	UK	2012	*Homo sapiens*	2	1114
**SAMEA7853291**	HBG-S2-62	Germany	NA	*Homo sapiens*	2	1114
**SAMN07258646**	11A1213CRGN021	Canada	2012	*Homo sapiens*	2	1114
**SAMN08398911**	MB_53	Spain	2013	*Homo sapiens*	2	1114
**SAMN09714554**	MB_177	Spain	2012	*Homo sapiens*	2	1114
**SAMN10249078**	AB_390	Pakistan	2016	NA	2	1114
**SAMN14605982**	Acinetobacter_baumannii_5707	Finland	NA	*Homo sapiens*	2	1114
**SAMN16357502**	FDAARGOS_1360	USA	NA	NA	2	1114
**SAMN19988021**	CNRAB1	France	2012	*Homo sapiens*	2	1114, 1841
**SAMD00030173**	SAMD00030173	Viet Nam	2011	*Homo sapiens*	2	136
**SAMEA4802589**	1231182		NA	NA	2	136

**SAMEA5226470**	4551f6d0-f9fe-11e8-b069-3c4a9275d6c8	United Kingdom	2018	*Homo sapiens*	2	136
**SAMN07258634**	07C16CRGN007A	Canada	2016	*Homo sapiens*	2	136
**SAMN15232059**	H73	China	2018	*Homo sapiens*	2	136
**SAMN19677632**	2021CK-01046	USA	2021	*Homo sapiens*	2	136
**SAMN19774564**	DETAB-C18	China	2019	*Homo sapiens*	2	136
**SAMN20674976**	2021CK-01178	USA	2021	*Homo sapiens*	2	136
**SAMEA2439285**	BAL062	NA	NA	*Homo sapiens*	2	136, 1851
**SAMN07258611**	01A15CRGN004	Canada	2015	*Homo sapiens*	2	451
**SAMN07450714**	GCID_GNCIT_00197_biosam	Thailand	2016	*Homo sapiens*	2	451
**SAMN08398923**	MB_85	Italy	2013	*Homo sapiens*	2	451
**SAMN08398938**	MB_114	Greece	2013	*Homo sapiens*	2	451
**SAMN12220285**	ACIN00232	USA	2018	*Homo sapiens*	2	451
**SAMN14669833**	AB1302	Belgium	2014	*Homo sapiens*	2	451
**SAMN14867459**	SS17M3332	Australia	2017	*Homo sapiens*	2	451
**SAMN19593362**	19-WSU-01-1039535	Turkey	2017	*Homo sapiens*	2	451
**SAMN27010291**	RAB97	Saudi Arabia	2019	*Homo sapiens*	2	451
**SAMN05601668**	KAB03	South Korea	2015	*Homo sapiens*	2	1809, 451
**SAMN03160611**	AMA474	Denmark	2013	*Homo sapiens*	2	218
**SAMN03470613**	AB210T1_Day23	USA	2013	*Homo sapiens*	2	218
**SAMN03699770**	M3AC14-8	Puerto Rico	2014	*Homo sapiens*	2	218
**SAMN07258696**	17A16CRGN001A	Canada	2016	*Homo sapiens*	2	218
**SAMN08398909**	MB_23	Spain	2012	*Homo sapiens*	2	218
**SAMN08398958**	MB_151	Italy	2015	*Homo sapiens*	2	218
**SAMN14669836**	AB1306	Kuwait	2014	*Homo sapiens*	2	218
**SAMN16541583**	6759_17	Hungary	2017	*Homo sapiens*	2	218
**SAMN27010204**	HAB30	Saudi Arabia	2019	*Homo sapiens*	2	218
**SAMN18202092**	Cl415	Lebanon	2017	*Homo sapiens*	2	2164, 218
**SAMN06117753**	GCID_ACINM_00198	USA	2012	*Homo sapiens*	2	417
**SAMN06117768**	GCID_ACINM_00213	USA	2012	*Homo sapiens*	2	417
**SAMN06117776**	GCID_ACINM_00221	USA	2013	*Homo sapiens*	2	417
**SAMN06117782**	GCID_ACINM_00227	USA	2013	*Homo sapiens*	2	417
**SAMN06461827**	GCID_ACINM_00306_biosample	USA	2012	*Homo sapiens*	2	417
**SAMN06461878**	GCID_ACINM_00315_biosample	USA	2012	*Homo sapiens*	2	417
**SAMN16351147**	ARLG_6361	USA	2018	*Homo sapiens*	2	417

**SAMN07520231**	5845	Mexico	NA	*Homo sapiens*	2	417
**SAMEA4802882**	1308782	NA	NA	NA	2	448
**SAMN25131632**	AB3-VUB	Belgium	2017	*Homo sapiens*	2	448
**SAMN16552564**	2020GO-00080	USA	2020	*Homo sapiens*	2	1806, 208
**SAMN14943601**	30111	China	2014	*Homo sapiens*	2	1806, 208
**SAMN07284119**	7847	Mexico	2008	*Homo sapiens*	2	1806, 208
**SAMEA1466048**	A96	Australia	2005	*Homo sapiens*	2	1806, 208
**SAMN06077192**	AF-673	USA	2008	*Homo sapiens*	2	1806, 208
**SAMD00165034**	NU-60	Japan	NA	NA	2	1806, 208
**SAMN03277095**	ORAB01	USA	2012	*Homo sapiens*	2	1806, 208
**SAMN03290686**	XH386	China	2017	*Homo sapiens*	2	1806, 208
**SAMN12287014**	19WIARLN007	USA	2019	*Homo sapiens*	2	208
**SAMEA1709711**	Ab183	Australia	2004	*Homo sapiens*	2	208
**SAMN15598653**	AB52LS	USA	NA	*Homo sapiens*	2	208
**SAMN19762490**	DETAB-E12	China	2019	NA	2	208
**SAMN12391534**	CFSAN093707	USA	2004	*Homo sapiens*	2	208
**SAMN06461788**	ABUH339	USA	2009	*Homo sapiens*	2	208
**SAMN23099163**	2021KU-00180	USA	2021	*Homo sapiens*	2	208
**SAMN14669889**	AB1396	Thailand	2013	*Homo sapiens*	2	208
**SAMEA1709803**	Ab208	Australia	2000	*Homo sapiens*	2	208
**SAMN18313887**	2021CK-00722	USA	2020	*Homo sapiens*	2	208
**SAMEA54070918**	214030705701	Netherlands	2012	*Canis familiaris*	2	350
**SAMEA54071668**	214031705301	Netherlands	2014	*Canis familiaris*	2	350
**SAMEA54072418**	214032504901	Netherlands	2014	*Canis familiaris*	2	350
**SAMN01828145**	NIPH-528	Netherlands	1982	*Homo sapiens*	2	350
**SAMN00761245**	OIFC338	USA	2003	NA	2	350
**SAMN14669779**	AB1135	USA	2009	*Homo sapiens*	2	350
**SAMN06117722**	ABUH462	USA	2009	*Homo sapiens*	2	350
**SAMN06117705**	ABUH390	USA	2009	*Homo sapiens*	2	350
**SAMN14669791**	AB1186	Romania	2011	*Homo sapiens*	2	350
**SAMN16541576**	2729_10	Hungary	2010	*Homo sapiens*	2	350
**SAMN06117778**	ABUH651	USA	2013	*Homo sapiens*	2	350

Table S3. Antibiotic susceptibility testing of study strains (publication ordering)

ST^{Pas}	ST^{Ox}	Strain	TIC	TIM	PIP	TZP	TZP (Sen.)	CAZ	FEP	MEM	IPM	GEN	TOB	AMK	KAN (MIC)	STR	APR (MIC)	CIP	LVX	MIN	TET (MIC)	SXT	COL (Sen.)
2532	New	ABO21-A001	27	27.8	25.8	25.9	8	22.4	27.4	29.8	36.7	23.5	22.4	27.1	4	17.3	8	30.4	30.7	36.6	1	24.1	0.5
1384	New	ABO21-A045	27.6	27.5	24.6	25.4	4	23.1	28.3	28.5	37.3	23.3	23	24.7	2	18.1	4	35.5	33.6	37.3	0.5	25.3	0.5
578	New	ABO21-A049	6	14.6	16.8	19.9	8	18.8	20.4	22.4	39.2	18.5	21.1	24.2	2	9.2	8	6	9.5	28.2	16	19.8	0.5
2247	New	ABO21-A051	27.5	28.4	25.9	25.8	4	23.1	29.5	30	38.5	22.3	22.2	25	1	17.2	4	32.6	33.2	38.3	0.5	24.2	0.5
2	350	ABO21-A002	6	6	11.5	19.3	16	20.1	22.7	21.2	32.7	17.2	19.4	21.6	40	6	16	6	13.2	27.8	16	21.9	0.5
2	350	ABO21-A003	6	6	9.9	17.3	>32	19	21.3	22.8	33.6	18	20.5	23.7	40	6	8	6	14.3	28.3	16	22.7	0.5
2	350	ABO21-A020	6	6	10.6	16.6	>32	20.5	22.5	21.9	33.4	17.3	20.9	24.4	40	6	8	6	14.8	29.2	16	24	0.5
2	350	ABO21-A022	6	6	9.8	17.1	>32	21.1	21.7	22.8	35	18.9	21.5	26.3	40	6	8	6	14.6	29.4	16	24.8	0.5
2	350	ABO21-A057	6	6	9.4	16.8	>32	20.7	22.7	22	34.1	19.3	20.5	25.6	40	6	8	6	14.2	30.5	16	24.8	0.5
2	350	ABO21-A059	6	6	13.1	19.8	8	21.2	23	21.6	34.8	18.4	21.8	26.2	4	6	8	6	14.9	29.8	16	24.8	0.5
2	350	ABO21-A061	6	6	10	17.3	32	18.8	21.9	21.3	34	17.9	20.1	23.8	40	6	8	6	14.4	29.5	16	23.9	0.5
2	350	ABO21-A063	6	6	9.2	15.7	>32	20.2	22.6	21.3	33.3	18.3	19.8	23.7	40	6	8	6	13.6	29	16	23	0.5
2	350	ABO21-A064	6	6	9.3	14.3	>32	19.3	21.6	21.3	33.5	18.1	21.1	25.6	40	6	8	6.4	14.6	29.3	16	24	0.5
2	208	ABO21-A058	6.5	12.7	10.5	12.5	>32	6	18.7	24.3	30.4	8.9	21.1	24.9	40	6	8	6	15.2	28.9	16	18.5	1

TIC: ticarcillin, TIM: ticarcillin-clavulanic acid, PIP: piperacillin, TZP: piperacillin-tazobactam, CAZ: ceftazidime, FEP: cefepime, MEM: meropenem, IMP: imipenem, GEN: gentamicin, TOB: tobramycin, AMK: amikacin, STR: streptomycin, CIP: ciprofloxacin, LVX: levofloxacin, MIN: minocycline, and SXT: trimethoprim-sulfamethoxazole were evaluated by disc diffusion assays. Sen.: MICs for piperacillin-tazobactam and colistin were obtained using Sensititre plates (Thermofisher) with increasing antibiotic concentration (indicated in µg/mL) and for TZP constant tazobactam concentration (4µg/L). MICs of colistin kanamycin, apramycin, and tetracycline were determined by broth microdilution are mentioned in µg/mL. Resistance levels based on EUCAST guidelines (available at https://www.sfm-microbiologie.org/wp-content/uploads/2022/05/CASFM2022_V1.0.pdf) are indicated by background color (resistant: red, susceptible: green, Intermediary: yellow). ST^{OX}350-ABO21 clonal strains are highlighted by a grey background color.

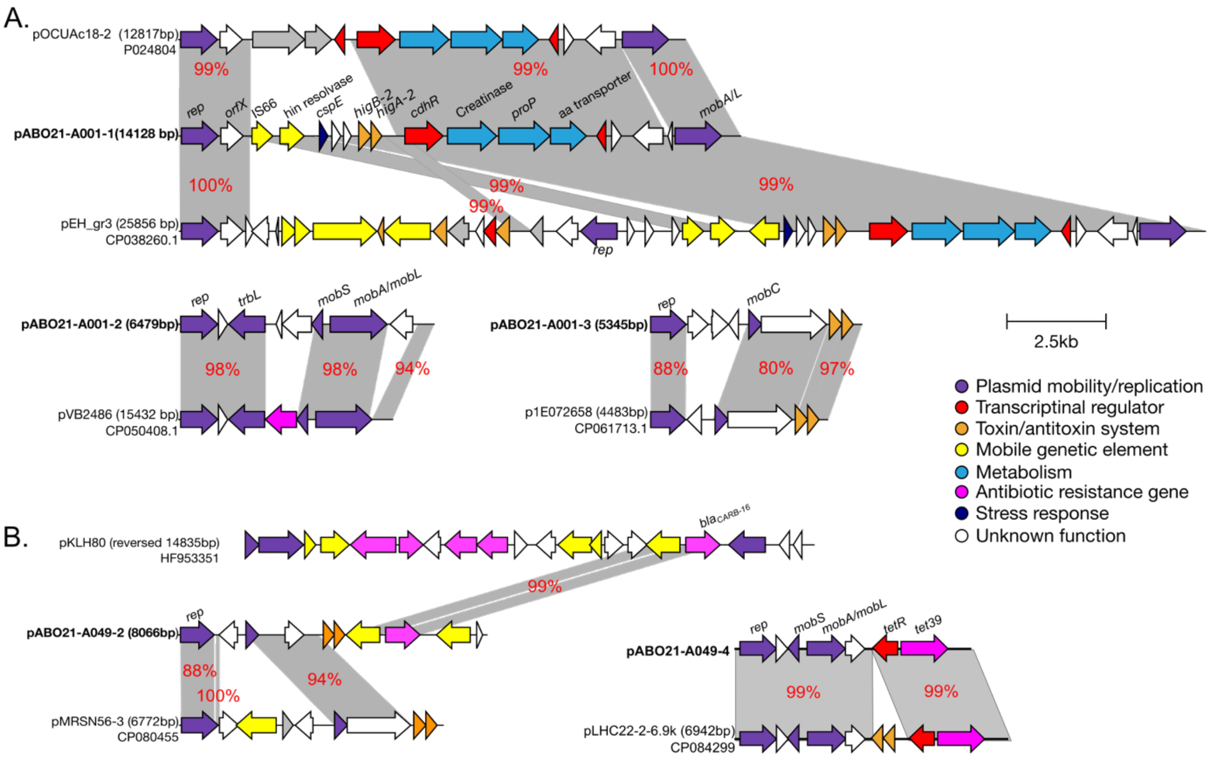

Figure S1. Structures of plasmids found in (A) ST^{Pas}2532 strain (ABO21-A001) and (B) ST^{Pas} 578 strain (ABO21-A049) isolated from animal ICU in comparison to those found in human isolates of *A. baumannii* (all but pKLH80) or in the permafrost bacterium *Psychrobacter maritimus* strain MR29-12 (pKLH80). Shades of grey indicate regions between 80% and 100% of nucleotide identity. Accession numbers are mentioned bellow each replicon name.

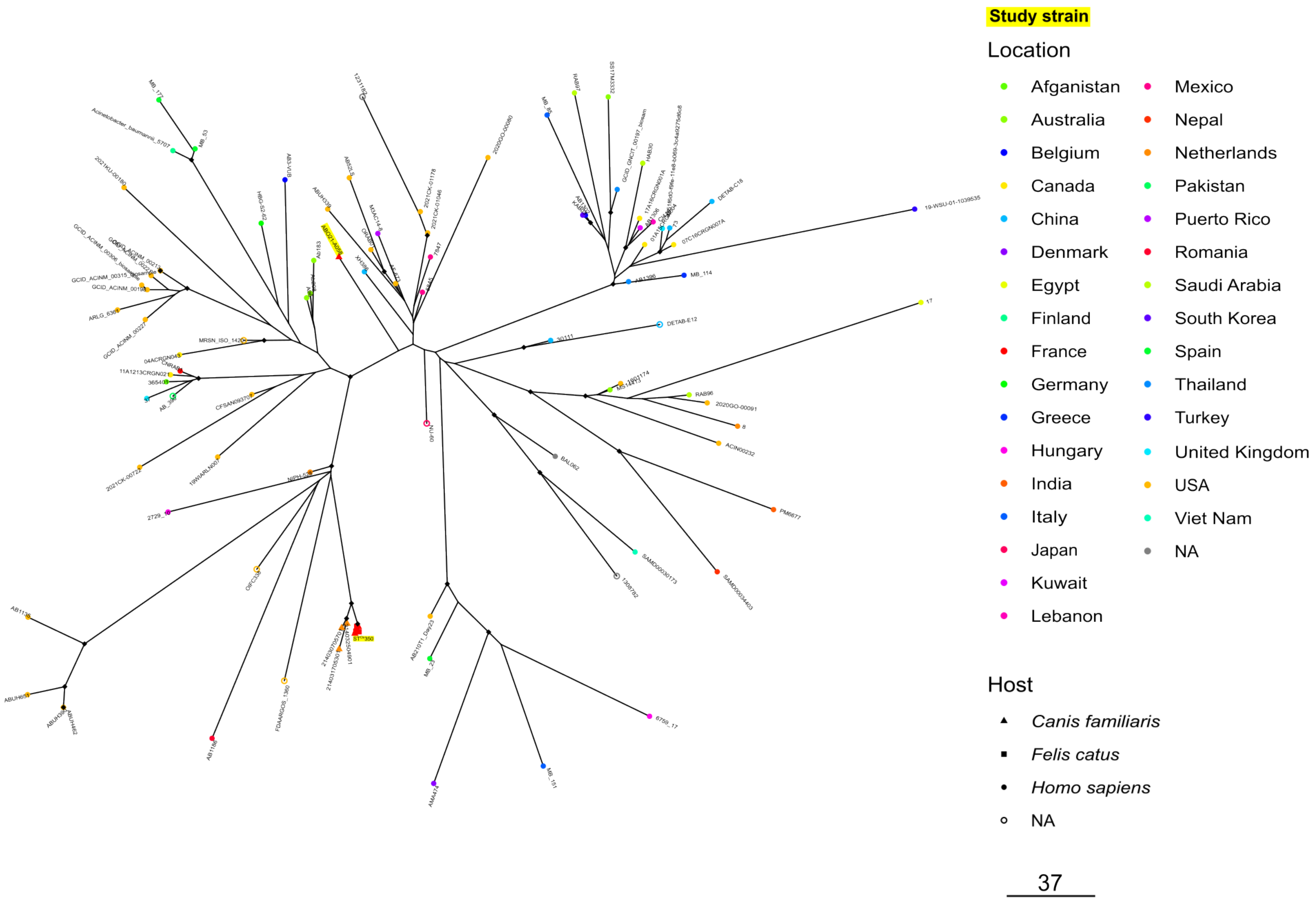

Figure S2. Phylogenetic proximity of animal ICU isolates with IC2 isolates. Maximum likelihood phylogenetic tree without recombination of 10 animal ICU isolates and 88 IC2 *A. baumannii* strains. ST Oxford is displayed as a heatmap. Location is displayed as tree tip-colored dots. Study strains are indicated by yellow shadowed labels. Host is displayed as tree tip shape. Bootstrap values equal to 100 are displayed as black diamonds. Scale unit: number of substitutions per genome. List of strains and their metadata are available in Table S2.

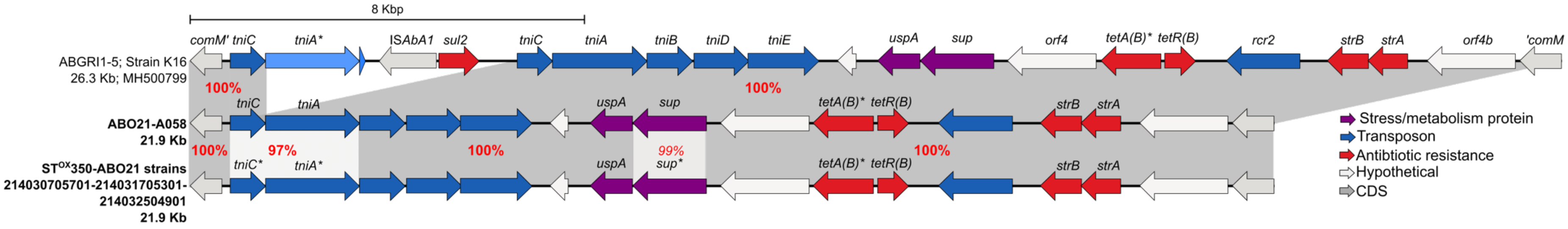

Figure S3. Structures of ABGRI1 resistance islands found in GC2 strains isolated from animal ICU in comparison to human isolate K16. Shades of grey indicate regions between 97% and 100% of nucleotide identity.

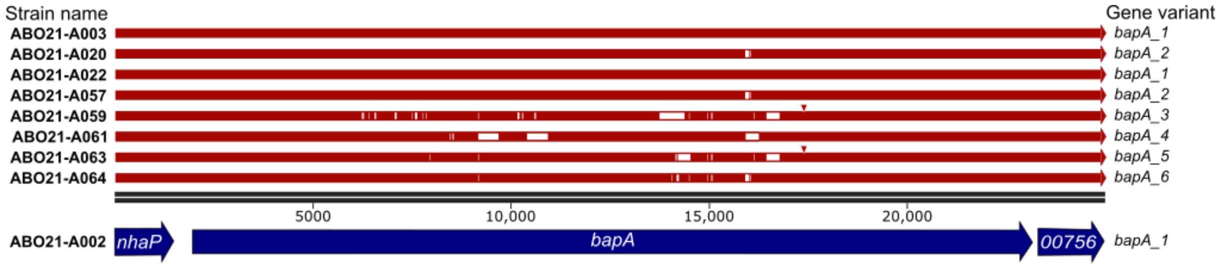

Figure S4. Comparative alignment of the *bapA* gene and its surrounding region in eight ST^{OX}350 isolates with the early ABO21-A002 isolate. Complete nucleotide sequence identity is highlighted by a continuous solid red line, while gaps and single nucleotide polymorphisms (SNPs) are represented by white regions. Insertions are denoted by triangle markers. Gene scale is indicated in nucleotide (figure adapted from a SnapGene alignment).
